# Global change scenarios trigger carry-over effects across life stages and generations of the intertidal limpet, *Siphonaria australis*

**DOI:** 10.1371/journal.pone.0194645

**Published:** 2018-03-21

**Authors:** Gustav M. Kessel, Nicole E. Phillips

**Affiliations:** School of Biological Sciences and Coastal Ecology Laboratory (VUCEL), Victoria University of Wellington, Wellington, New Zealand; University of Connecticut, UNITED STATES

## Abstract

For organisms with complex life histories, carry-over effects (COEs) can manifest between life stages, when conditions experienced by one stage influence the next, as well as trans-generationally, when the parental environment affects offspring. Here we used multiple global change-associated stressors to examine both forms of COE simultaneously in an intertidal limpet with mixed development (i.e. planktonic larvae hatch from benthic egg masses). Adult *Siphonaria australis* were subjected to four treatments over four weeks: an ambient control, a treatment featuring elevated water temperature (25°C) and UVB (1.7 W m^-2^), a copper pollution treatment (5.0 μg L^-1^), and a treatment incorporating all three stressors. Egg masses laid by these adults were then redistributed among the same four treatments (producing 16 adult-to-egg treatment histories) and stressed until hatching. Finally, hatching larvae were reared under ambient conditions for 24 days. While adult survivorship was unaffected by treatment, embryonic viability in egg masses responded strongly to egg mass treatment, as well as parental stress exposure, therefore displaying trans-generational COEs. These trans-generational COEs interacted with COEs originating in egg masses to produce highly context-dependent hatching sizes and larval growth. This demonstrates that the performance of a given organism at a given time reflects not only conditions experienced during embryonic development, but also those of the parental generation, and suggests that COEs play an important but underestimated role in responses to global change scenarios.

## Introduction

The early life stages of marine organisms can be highly sensitive to stress, especially developing embryos encapsulated within benthic egg masses, which cannot escape prevailing conditions [1, 2, 3]. Further, the survivors of direct developmental stress may continue to suffer from its impacts long after exposure has ceased, since the conditions experienced during one life stage can significantly influence the performance or characteristics of subsequent, morphologically and ecologically distinct stages through carry-over effects (COEs) [4, 5, 6]. For example, exposure to sub-optimal levels of salinity [7], temperature [8], nutrition [9, 10, 11] and pollution [12] in larval stages of various marine invertebrates can produce legacies of poor juvenile survival and growth that in some cases extend even into adulthood and reduce fitness. Additionally, the capability for parental stress to affect offspring through trans-generational COEs is now widely recognised [13]. Trans-generational COEs, or non-genetic parental effects, are a poorly understood mechanism through which environmental factors occurring in the parental generation can manifest in changes to offspring phenotype that cannot be attributed to the genotype or environment of offspring themselves [14, 15, 16, 17, 18, 19]. This process has been regarded as fundamental to evaluating the persistence of marine invertebrates in the face of ongoing global change [20].

Global change is increasingly impacting coastal marine systems [21, 22], including the rocky intertidal zone [23]. Rock pools are dynamic environments that naturally experience dramatic fluctuations in several physico-chemical parameters over diel, tidal and seasonal time scales [24]. Their inhabitants may be especially vulnerable to additional anthropogenic influences, which may augment the already stressful and variable conditions to which they are routinely exposed, causing tolerance thresholds to be exceeded [25, 26]. Surface levels of solar UVR (wavelengths of 280-400nm) continue to increase in spatially uneven ways [27] and short wave-length UVB (280-315nm) in particular, can have a myriad of deleterious effects on aquatic organisms, including increased mutation rates, elevated disease risk and impaired or abnormal development (reviewed by [28]). In rock pools, high UVB levels often occur in concert with high water temperature, particularly when low tides coincide with hot, clear weather during summer, a circumstance that is expected to become more common with ongoing climate change [21, 29]. High temperatures of over 30°C may be reached in rock pools even at temperate latitudes [30], which can lead to protein damage and adversely affect vital physiological processes such as membrane fluidity and organ function [31].

In addition to climate change, growing coastal human populations cause marine organisms to come into more frequent contact with pollutants, and it is likely that this will play an increasing role in future intertidal stress regimes. Among the cocktail of contaminants entering coastal waters, metals are considered major pollutants, and of these copper (Cu) is among the most common and concerning [32, 33]. Urban and agricultural runoff are common sources of Cu pollution [34], the accumulation of which can be severe after rain [35]. Cu is one of the most toxic metals [36], and exposure can have an array of severe physiological and behavioural effects, including immune system suppression in mussels [37], heart rate reduction in gastropods [38], and reduced clamping tenacity in limpets [39], as well as impaired reproduction, development and growth in organisms across a wide range of taxa [40]. The toxicity of heavy metals like Cu is highly variable and depends to a considerable extent on other environmental factors, including UVB, and water temperature [41].

Although awareness of synergistic stressor interactions and the use of multifactorial designs is increasing [42], until recently most global change-related studies have used only a single stressor [43]. Moreover, comparatively little attention has been given to the propagation of stress through the often dramatically disparate life stages of marine organisms, how its effects are passed through to successive generations, or how trans-generational COEs interact with those occurring between life stages, especially in a global change context. As a result, a given organism may be influenced by agents of global change through a far wider range of avenues than commonly acknowledged, extending from its own life history to that of its progenitors.

*Siphonaria australis* (Quoy and Gaimard 1833) is a small pulmonate limpet native to New Zealand that deposits benthic, gelatinous egg masses in intertidal rock pools, particularly during summer, which hatch after ~1–2 weeks into feeding veliger larvae [44]. Egg masses are thus often exposed to extreme levels of UVB and water temperature, leading to direct mortality in developing embryos [2], as well as COEs in the form of reduced larval growth and survival when larvae are subjected to further stress [45]. Here, a laboratory experiment was used to examine how the effects of exposure to stressors relating to climate change (high water temperature and UVB), as well as compounding factors (Cu pollution), are carried over not only between these life stages (embryos encapsulated in egg masses to planktonic larvae) but also between generations (from parent to offspring). Through this approach, we aimed to more fully elucidate the role of COEs in the face of ongoing global change.

## Materials and methods

### Adult treatments

Adult *S*. *australis* (n = 120) were collected during low tide from rock pools in the Wellington region of New Zealand (Houghton Bay, 41°20’51.9"S 174°47’29.0"E) in January 2015. They were transported to the Victoria University of Wellington Coastal Ecology Laboratory (VUCEL) in seawater, and maintained in twelve 10L buckets, each containing ten specimens and a hose delivering a continuous supply of filtered seawater (FSW, 15μm mesh size). Mean shell length per bucket ranged from 15 to 15.3 mm. Each bucket was furnished with rocks collected at the same site, providing ~5m^2^ of surface area on which limpets could graze algal films. Prior to experimental treatment, limpets were acclimated in this set-up for four weeks. During this time adequate feeding was confirmed by the presence of faecal material in buckets.

Following acclimation, limpets were subjected to four experimental treatments for four weeks. Each treatment consisted of three replicate buckets. Treatments included a no stress control with ambient levels (in laboratory) of water temperature and UVB and no added Cu (“No Stress” = ~16°C; 0.087 W m^-2^; 0 μg L^-1^ Cu), a treatment with elevated water temperature and UVB but no added Cu (“Temp/UV” = 25°C; 1.7 W m^-2^; 0 μg L^-1^ Cu), a pollution treatment with added Cu only (“Cu” = ~16°C; 0.087 W m^-2^; 5.0 μg L^-1^), and an extreme treatment with elevated levels of all stressors (“Temp/UV/Cu” = 25°C; 1.7 W m^-2^; 5.0 μg L^-1^ Cu). Note that water temperature and UVB were elevated together because both stressors are derived from sun exposure and unlikely to occur separately in rock pools in summer when egg masses are deposited [29]. Limpets were exposed to these treatments for 4 hr each day, to approximate the duration of low tide, at which time they would be most vulnerable to these stressors in rock pools.

During daily treatment exposure, buckets were disconnected from their seawater supply. Elevated temperature treatments were heated to 25°C using aquarium heaters. For buckets treated with elevated UVB, daylight bulbs were replaced with Philips TL40W/03RS UV bulbs, which were wrapped in clear PVC foil to eliminate extremely short wavelength UVC. This ensured that the amount of UVB reaching the water surface in buckets was 1.7 W m^-2^, the mean daily maximum value for Wellington rock pools at low tide in summer, as measured on several cloudless days in January 2015 [46]. By comparison, UVB inside VUCEL was 0.08 W m^-2^. UVB was measured using a Skye SpectroSense2+ UVB sensor radiometer. Finally, buckets treated with elevated Cu were dosed using a copper stock solution (copper sulphate: CuSO_4_.5H_2_O) to achieve a concentration of 5.0 μg L^-1^, a maximum common reading for Wellington seawater [35]. Food-grade Teflon® buckets were used to ensure that no Cu was absorbed and that limpets would not be exposed to Cu residue outside of treatment times [47]. Buckets were reconnected to the seawater supply and returned to ambient conditions after the four hours of stress exposure elapsed. After four weeks of treatment, the number of surviving adults per bucket was recorded.

### Egg mass treatments

Egg masses were collected on the last day of adult treatments (laid after that day’s stress exposure), and on the subsequent two mornings (laid during the night). Immediately on collection, four egg masses were obtained from each of the twelve buckets, and transferred to dishes (one egg mass per dish containing 100 ml of FSW) for further experimentation. *Siphonaria*, including *S*. *australis*, generally take 24–48 hr to reach gastrulation [48, 49]. Thus, because egg masses were collected within 18 hr of deposition, embryo developmental stage at the start of their experimental treatments likely ranged from early cleavage to early gastrulation.

The four egg masses taken from each replicate bucket were assigned to each of four egg mass treatments, which comprised the same four treatments as previously used for the adults. This equated to 16 possible adult-to-egg mass (written as “adult→egg mass”) treatment histories. Each of these 16 treatment histories consisted of three replicate dishes (total n = 48 dishes). All egg masses were stressed for 4 hr per day for up to two weeks, or until hatching occurred. Note that because each egg mass was transferred to egg mass treatments immediately upon being found, egg masses that were transferred on the last day of adult stress exposure began their treatment earlier than those transferred on the subsequent mornings, which experienced identical treatment duration administered in parallel but offset by one or two days. Water in the dishes was changed daily after the 4 hr stressing period.

Egg masses that failed to hatch by the end of the two-week egg mass stress period, as well as ~5mm pieces removed from each successful egg mass upon hatching, were used to estimate the percentage of viable embryos per egg mass. Sections were taken from each and examined under 100x magnification on a slide using a compound microscope. Similar to methods used by [2], 50 embryos from each section were scored as viable (i.e. normally developed embryos) or inviable (i.e. severely abnormal such as deformed blastulae).

### Rearing of larvae

Upon hatching, 500 swimming larvae were individually transferred via pipette from each egg mass dish into a jar with 800 ml FSW. Because not all egg masses hatched, only 11 of the 16 possible treatment histories were represented, and because 18 egg masses failed, 30 rather than 48 jars were used. Note that some of the treatment histories included only two rather than three replicates as a result of hatching failures.

All larvae were reared under ambient conditions (water temperature at ~16°C, UVB exposure at 0.087 W m^-2^, and 0 μg L^-1^ of Cu) so that any COEs manifesting in larvae could be clearly identified. Additionally, cetyl alcohol granules were sprinkled liberally across the water surface in jars, where they floated and acted to break up the surface tension, which may otherwise entrap larvae and cause mortality [50]. Water changes (80%) were carried out every three days. Larvae were fed after each water change with a 50:50 mixture of two algal species, *Isochrysis galbana* and *Pavlova lutheri* (cultured at VUCEL), at a concentration of 20,000 cells ml^-1^. All jars were stirred daily to re-suspend algal cells and hinder the formation of flocculent detritus. Larvae were reared for 24 days (after which larvae began to reach competence, a point at which they are able to settle to the substratum and metamorphose into their juvenile stage), but no effort to induce settlement was made. Upon hatching (day 0) and at 24 days, a random sample of 30 larvae was taken from each container. These were placed on a slide and their shell lengths were measured using a compound microscope at 100x magnification.

### Data analysis

All statistical analyses were carried out using SPSS version 23 software (IBM Corp, Armonk, NY, USA). All relevant assumptions were met unless stated otherwise, and data was not transformed. Separate binomial logistic regressions were carried out to examine the effect of adult treatment (fixed factor with 4 levels) on adult survivorship using 120 adults from 12 buckets (bucket included as random factor) scored as survived or not survived, and the effects of adult and egg mass treatments (both fixed factors with 4 levels each) on embryo viability using 2,400 embryos from 48 egg mass dishes scored as viable or inviable. Due to departures from normality and homogeneity of variance, a Kruskal-Wallis test was used to examine the effect of total treatment history (11 levels; treatment histories in which no egg masses hatched were excluded) on hatching time (number of days from being laid to hatching), using 30 successfully hatched egg masses.

To examine the effect of adult and egg mass treatments on larval size over time, a three-way mixed ANOVA (GLM repeated measures procedure) was carried out using adult treatment (4 levels), egg mass treatment (3 levels; extreme stress excluded because no larvae from this egg mass treatment hatched successfully), and time (2 levels; day 0 hatching time and day 24) as fixed factors and larval container as a random factor. Adult and egg mass treatments were considered as between-subject factors, and time as a within-subject factor. For this analysis, some data departed from normality and homogeneity of variance, but because the test is considered robust to these violations and transformation failed to remedy this, the test was carried out nonetheless using untransformed data [51]. The assumption of sphericty was also violated, and accordingly, Greenhouse-Geisser values were used to interpret the statistical significance of the three-way interaction. To further explore this interaction, two-way ANOVAs were carried out (with adult and egg mass treatment as fixed factors with 4 and 3 levels respectively) for each of the two time points, to test for two-way interactions between adult and egg mass treatment, their main effects, and pairwise comparisons. As part of the GLM procedure, pairwise comparisons were carried out between treatment histories that either shared the same adult treatments but differed in egg mass treatment (significant differences indicating COEs between life stages), or between treatment histories that shared the same egg mass treatment but differed in adult treatment (significant differences indicating trans-generational COEs). Pairwise comparisons between treatment histories that differed in both adult and egg mass treatment were not carried out as these offer no indication of whether COEs occurred. All alpha levels were Bonferroni-adjusted where appropriate.

## Results

### Adult survivorship

Adult treatment did not significantly affect adult survival likelihood (*r*^*2*^ = 0.216; *X*^*2*^(11) = 18.916; *P* = 0.063), but survivorship among adults in the extreme stress treatment that included all three stressors exhibited a trend of being slightly lower than the others (mean number of survivors out of 10: No Stress = 8; Cu = 8.3; Temp/UV = 7.3; Temp/UV/Cu = 6.3).

### Embryo viability

**Fig 1:**
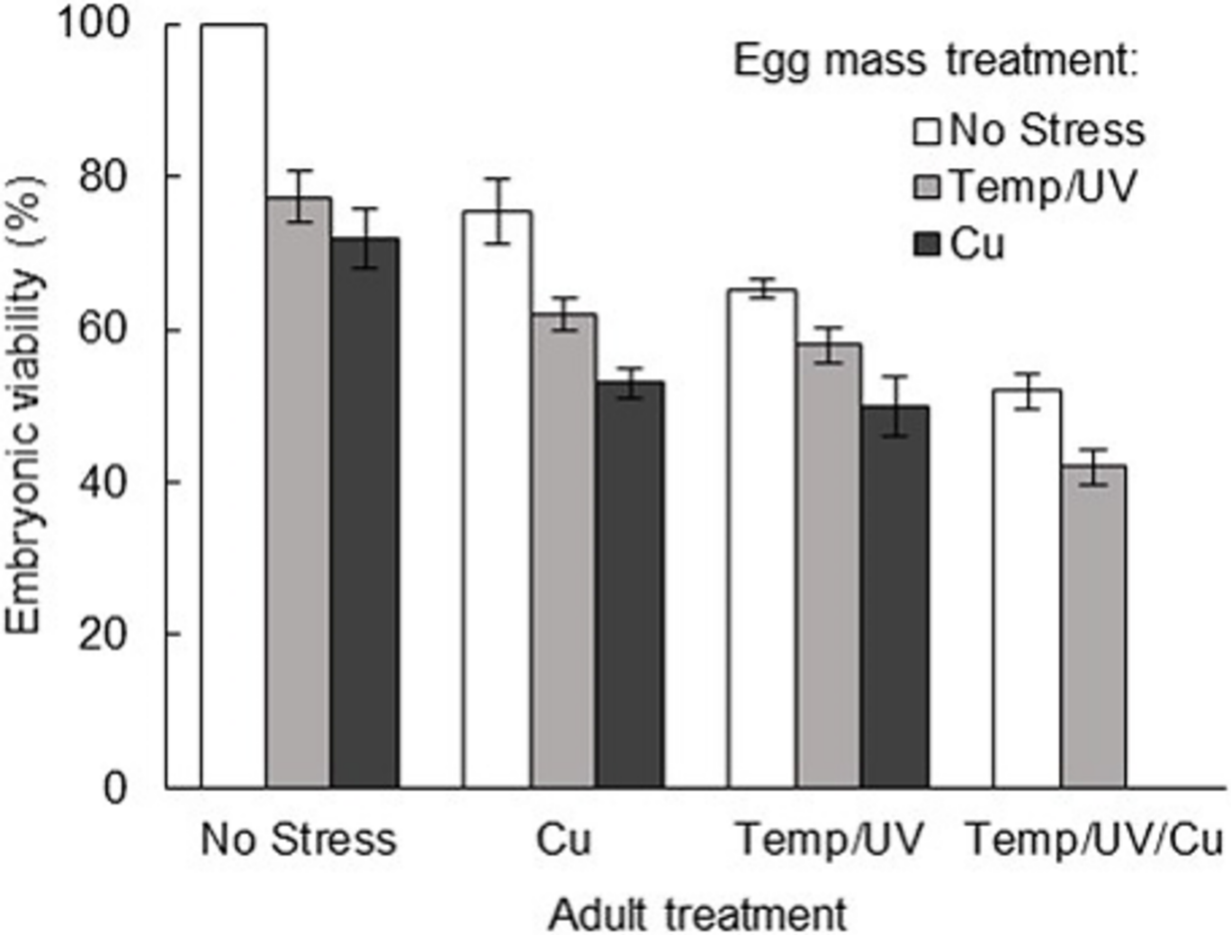
Embryonic viability of *S*. *australis* in response to experimental treatments of parents and egg masses. Mean embryonic viability from egg masses originating from different combinations of parental and egg mass stress treatments (± 95% CI, *n* = 48 egg masses). All bars showing egg masses treated with Cu display means calculated from successfully hatching egg masses only, and were therefore calculated from two rather than three replicate egg masses due to hatching failures in these treatment histories.

Embryo viability was strongly affected by parental stress as well as stress experienced in the egg mass. The logistic regression model was significant (*r*^*2*^ = 0.535; *X*^*2*^(6) = 1211.535; *P* < 0.001), with significant main effects of both adult and egg mass treatment, but no significant interaction (Table 1). Odds ratios (Table 1) show that embryonic viability decreased according to egg mass treatment in the order No Stress > Temp/UV > Cu > Temp/UV/Cu, and according to adult treatment in the order No Stress > Cu > Temp/UV > Temp/UV/Cu, giving the step-like pattern in Fig 1. Trans-generational COEs were therefore found, where adult stress resulted in lower likelihoods of viability in offspring. None of the egg masses subjected to extreme stress (Temp/UV/Cu) hatched, regardless of parental treatment, and only two out of three egg masses hatched for the treatment histories where egg masses were subjected to copper. All egg masses that failed to hatch contained 0 viable embryos. Among successfully hatched egg masses, no significant difference in hatching time was found between treatment histories (Kruskal-Wallis Chi-square = 11.969; *df* = 10; *P* = 0.287), and hatching tended to occur 6–9 days after being laid, regardless of treatment history.

**Table 1:** Logistic regression predicting likelihood of embryonic inviability of *S*. *australis* based on adult and egg mass (abbrev. egg) treatment.

Predictor	β	*SE* β	Wald’s χ^2^	*df*	*P*	Odds Ratio
Adult (main effect)			187.011	3	**<0.001**	
Adult Cu	0.966	0.159	36.857	1	**<0.001**	2.627
Adult Temp/UV	1.226	0.159	59.306	1	**<0.001**	3.406
Adult Temp/UV/Cu	2.252	0.166	183.186	1	**<0.001**	9.508
Egg (main effect)			289.344	3	**<0.001**	
Egg Cu	2.160	0.141	233.051	1	**<0.001**	8.670
Egg Temp/UV	0.688	0.133	26.786	1	**<0.001**	1.989
Egg Temp/UV/Cu	7.839	1.008	60.471	1	**<0.001**	2538.640
Adult x Egg			2.061	9	0.990	
Constant	-2.255	0.155	211.791	1	**<0.001**	0.105

For odds ratios, the listed adult or egg treatment is compared to its respective control (i.e. No Stress).

### Larval size

Stress regimes experienced by both parents and egg masses had complex effects on both larval size at hatching, and larval size 24 days after growing in similar ambient conditions. A significant three-way interaction between adult treatment, egg mass treatment and time was found (*F*_5, 889_ = 42.036; *P* < 0.001; partial η^2^ = 0.191), and there were statistically significant two-way interactions between adult and egg mass treatments at day 0 (*F*_5, 889_ = 103.037; *P* < 0.001) and day 24 (*F*_5, 889_ = 12.364; *P* < 0.001). Jar had no significant effect. Using a Bonferroni-adjusted alpha level of 0.017, there was a significant main effect of adult treatment on larval size at all levels of egg mass treatment at both time points (Table 2). There was also a significant main effect of egg mass treatment on larval size under all levels of adult treatment at both time points (using a Bonferroni-adjusted alpha level of 0.0125), except for larvae produced by extremely stressed adults at day 0, and larvae produced by unstressed adults at day 24 (Table 2).

**Table 2:** Main effects of three-way mixed ANOVA examining the effect on *S*. *australis* larval shell length of adult treatment at each level of egg mass (abbrev. egg) treatment, and of egg treatment at each level of adult treatment, at two time points.

Day	Independent	Dependent	*df*	*F*	*P*
**0**	Adult Treatment	Egg No Stress	3, 889	32.266	**<0.001**
		Egg Cu	2, 889	147.397	**<0.001**
		Egg Temp/UV	3, 889	52.700	**<0.001**
	Egg Treatment	Adult No Stress	2, 889	185.241	**<0.001**
		Adult Cu	2, 889	11.824	**<0.001**
		Adult Temp/UV	2, 889	149.351	**<0.001**
		Adult Temp/UV/Cu	1, 889	4.656	0.031
**24**	Adult Treatment	Egg No Stress	3, 889	15.289	**<0.001**
		Egg Cu	2, 889	18.177	**<0.001**
		Egg Temp/UV	3, 889	6.749	**<0.001**
	Egg Treatment	Adult No Stress	2, 889	0.527	0.591
		Adult Cu	2, 889	19.539	**<0.001**
		Adult Temp/UV	2, 889	50.149	**<0.001**
		Adult Temp/UV/Cu	1, 889	13.561	**<0.001**

**Fig 2:**
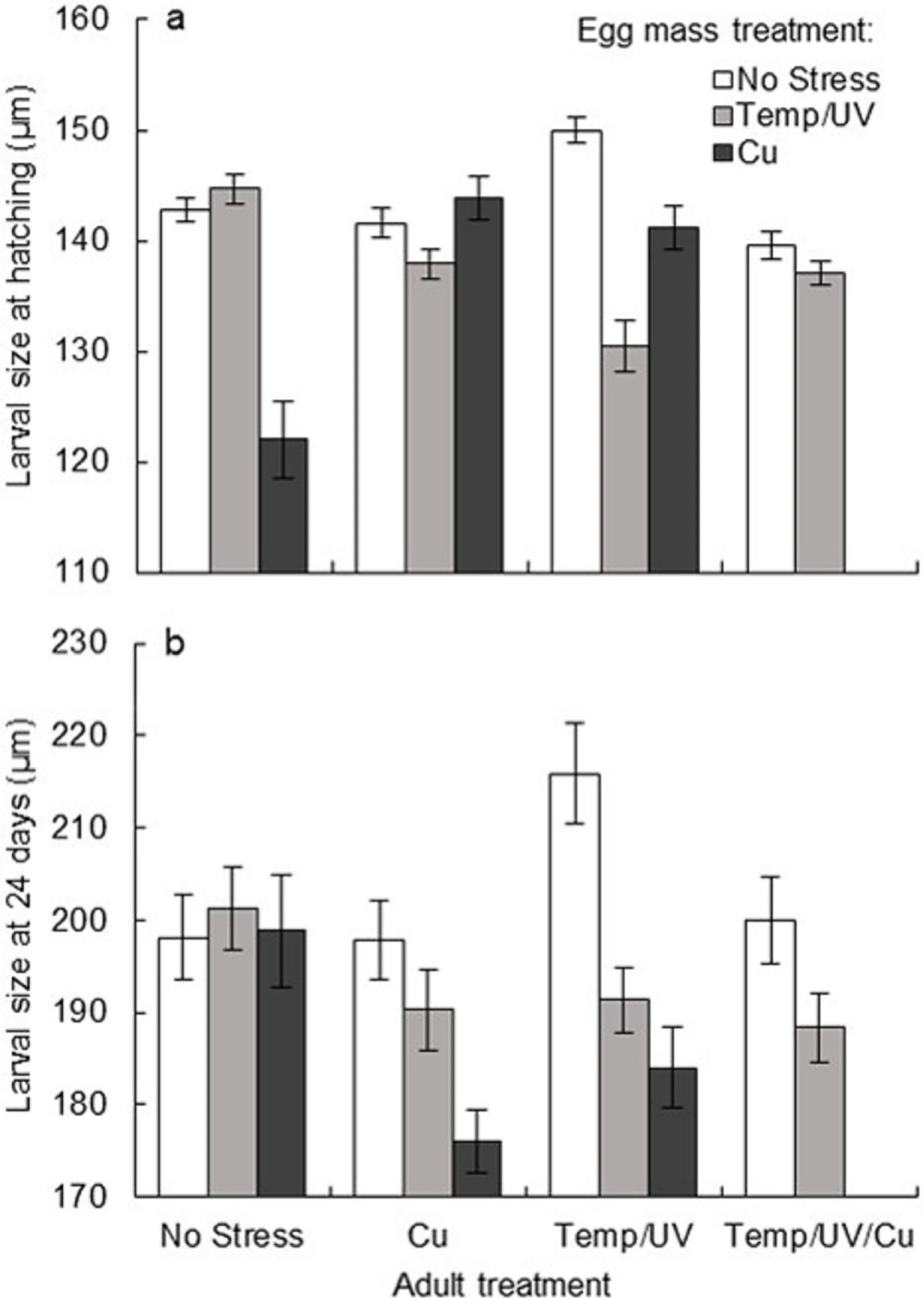
Complex effects of both parental and egg mass treatment on larval size. Mean shell length of *S*. *australis* larvae in response to experimental adult and egg mass treatment (± 95% CI, *n* = 30 jars of larvae containing the hatchlings of one egg mass each), at a) day 0 (hatching), b) 24 days.

Mean hatching size ranged from ~122μm in larvae from the No Stress→Cu treatment history, to ~150μm in larvae from Temp/UV→No Stress. Most larvae hatched at a mean size of 140–145μm (Fig 2A). Overall, there was a high degree of variation in hatching size which was similarly affected by both forms of COEs, with pairwise comparisons showing seven instances of COEs between life stages (from embryo to larva) to have occurred (Table 3), and ten instances of trans-generational COEs (Table 4). Both forms of COEs were most frequently observed at hatching. Mean size at 24 days ranged from ~175μm in Cu→Cu, to ~217μm in Temp/UV→No Stress, with larvae from most other treatment histories ranging from ~190μm ~200μm (Fig 2B). Here, larvae were affected by COEs between life stages in five instances, and by trans-generational COEs in seven instances (Tables 3 and 4). COEs between life stages diminished in frequency over time (seven at 0 days, five at 24 days), and so did trans-generational COEs (ten at 0 days, seven at 24 days), while trans-generational COEs were more frequent than those between life stages at both time points (Tables 3 and 4).

**Table 3:** Pairwise comparisons of *S*. *australis* larval shell lengths between treatment histories sharing the same adult treatment, but differing in their egg mass treatments, at two time points (performed as part of GLM procedure).

Day	Treatment History I	Treatment History J	*P*
**0**	No Stress→No Stress	No Stress→Cu	**<0.001**
	No Stress→Cu	No Stress→Temp/UV	**<0.001**
	Cu→No Stress	Cu→Temp/UV	**0.004**
	Cu→Cu	Cu→Temp/UV	**<0.001**
	Temp/UV→No Stress	Temp/UV→Cu	**<0.001**
	Temp/UV→No Stress	Temp/UV→Temp/UV	**<0.001**
	Temp/UV→Cu	Temp/UV→Temp/UV	**<0.001**
**24**	Cu→No Stress	Cu→Cu	**<0.001**
	Cu→Cu	Cu→Temp/UV	**<0.001**
	Temp/UV→No Stress	Temp/UV→Cu	**<0.001**
	Temp/UV→No Stress	Temp/UV→Temp/UV	**<0.001**
	Temp/UV/Cu→No Stress	Temp/UV/Cu →Temp/UV	**<0.001**

Only those comparisons significant at the Bonferroni-adjusted alpha level of 0.005 are displayed. These differences are indicative of COEs between life stages (from egg mass to larva).

**Table 4:** Pairwise comparisons (simple-simple) of *S*. *australis* larval shell lengths between treatment histories sharing the same egg mass treatment, but differing in their adult treatments, at two time points (performed as part of GLM procedure).

Day	Treatment History I	Treatment History J	*P*
**0**	No Stress→No Stress	Temp/UV→No Stress	**<0.001**
	Cu→No Stress	Temp/UV→No Stress	**<0.001**
	Temp/UV→No Stress	Temp/UV/Cu→No Stress	**<0.001**
	No Stress→Cu	Cu→Cu	**<0.001**
	No Stress→Cu	Temp/UV→Cu	**<0.001**
	No Stress→Temp/UV	Cu→Temp/UV	**<0.001**
	No Stress→Temp/UV	Temp/UV→Temp/UV	**<0.001**
	No Stress→Temp/UV	Temp/UV/Cu→Temp/UV	**<0.001**
	Cu→Temp/UV	Temp/UV→Temp/UV	**<0.001**
	Temp/UV→Temp/UV	Temp/UV/Cu→Temp/UV	**<0.001**
**24**	No Stress→No Stress	Temp/UV→No Stress	**<0.001**
	Cu→No Stress	Temp/UV→No Stress	**<0.001**
	Temp/UV→No Stress	Temp/UV/Cu→No Stress	**<0.001**
	No Stress→Cu	Cu→Cu	**<0.001**
	No Stress→Cu	Temp/UV→Cu	**<0.001**
	No Stress→Temp/UV	Cu→Temp/UV	**0.003**
	No Stress→Temp/UV	Temp/UV/Cu→Temp/UV	**<0.001**

Only those comparisons significant at the Bonferroni-adjusted alpha level of 0.003 are displayed. These differences are indicative of trans-generational COEs.

## Discussion

The current study simultaneously examined two types of COEs, those that can occur across generations as well as between life stages. We found that these two types of COEs often interacted to form highly context-dependent legacies of mostly impaired performance in *S*. *australis* larvae in response to multiple stressors. This research highlights the important role of COEs, as determined by the previous history of individuals and their progenitors, in the responses of organisms to global change scenarios.

Adult survival was largely unaffected by the stress levels imposed on them. This was not surprising, since a previous study has demonstrated that adult *Siphonaria* limpets are relatively resistant to even continuous exposure to Cu over several days [52]. Here the exposure times were limited to four hours per day. Moreover, other Cu-sensitive marine taxa such as copepods, clams and flounder have failed to show significant mortality in response to concentrations as high as 28–39 μg L^-1^ if exposed for less than 96 hours (reviewed by [53]). Additionally, rock-pool dwelling gastropods in general are relatively robust to high temperatures [54, 55] and UVR exposure [56]. However, in mussels for example, synergistic interactions of Cu and other stressors, particularly high water temperature, are known to reduce the exposure time necessary for mortality to occur [57], which may also explain the trend of reduced survival among adults exposed to the extreme stress treatment, which combined Cu, temperature and UVB. Exposure times longer than four weeks may have yielded different results, perhaps, due to the significant effects of recent thermal history and pre-conditioning among intertidal organisms enabling adults to better withstand temperature stress [58], or conversely, by increasing the severity of Cu or UV stress or synergistic interactions. Nevertheless, while adult *S*. *australi*s experienced minimal effects themselves, their exposure to stress invoked trans-generational COEs that manifested in developing offspring, and continued to interact with stress experienced as encapsulated embryos in egg masses to form highly context-dependent size and growth patterns throughout the planktonic larval stage.

Egg masses were much more vulnerable to stress than adults. Egg mass Cu exposure in particular, was the main factor that either limited hatching success when it was the only stressor, or prevented hatching entirely when coupled with elevated temperature and UVB under the extreme stress treatment, regardless of adult treatment. This is consistent with the general literature, which documents taxonomically wide-spread vulnerability to metal pollution at early life stages [40, 53]. Moreover, the failure of egg masses exposed to all stressors (Temp/UV/Cu) also indicates developmental synergism between climate change stress (high temperature and UVB) and pollution (Cu) that is similar to previously documented responses in other taxa [43]. Since all failed egg masses contained only inviable blastulae, Cu is likely to have interfered with cell division and entirely halted proper embryonic development [36].

Embryonic viability in *S*. *australis* was also significantly lower in egg masses exposed to the Temp/UV treatment than in unstressed egg masses, reaffirming the results of [2] and [45], who also documented reduced embryonic viability in *S*. *australis* egg masses with temperature and UVB stress. Because encapsulated embryos of intertidal gastropods generally exhibit high tolerance to elevated water temperatures [59], which may be achieved through the presence of thermally protective proteins [1], this result is most likely a consequence of the UVB component of this treatment, or a synergistic interaction between UVB and high temperature. UVB is generally highly deleterious to developing embryos, capable of causing deformity and mortality at similar levels to those incorporated here [60], including in *S*. *australis* [2].

Embryonic viability was also mediated by parental experience, tending to be higher in egg masses laid by unstressed adults than those of Cu-stressed adults, which was in turn higher than in those laid by Temp/UV-stressed adults, with adults under extreme stress producing the lowest viability. This trans-generational influence was most severe for Cu-treated egg masses, which were a complete failure if their parents were exposed to the extreme stress treatment. Under all other adult treatments, two of three Cu-treated egg masses hatched. This difference in offspring hatching success can only be attributed to the different treatments their parents experienced, evidence for a trans-generational COE in which extreme stress in adults prevented hatching when offspring encountered Cu and highlighting an interaction between parental and offspring experiences. The other Cu-treated egg masses hatched at the same rate and showed similar percentages of viability regardless of whether their parents experienced Cu stress or not, showing that adults did not convey any evident resistance to offspring. This contrasts with several previous studies of COEs across diverse taxa in which parents exposed to various toxicants produce offspring that are themselves at least initially resistant [61, 62], including to Cu in gastropods [63], but supports studies that have found reduced offspring quality [64].

The very low viability of eggs laid by extremely stressed adults may be best explained by the aforementioned synergistic stressor interaction in adults under the Temp/UV/Cu treatment. The deleterious effects of pollutants, including Cu, are readily enhanced by UVR through non-lethal phototoxicity (reviewed by [65])and by high water temperature (reviewed by [66]), either of which may have interfered with fertilisation or the formation of gametes in such a way so as to produce offspring of lower quality. Such impairments to reproduction have been observed in response to numerous metals, including Cu [40]. Although the effects of UVR and temperature cannot be separated, since they were combined within a single treatment, the results nonetheless indicate that synergistic trans-generational effects, previously documented only in plants [67, 68], have indeed taken place in one form or another.

In larvae, responses were more complex. There were clear COEs between the benthic egg mass and planktonic larval stages, as larvae from egg masses treated with Cu or Temp/UV generally hatched at smaller sizes than from unstressed egg masses for each parental treatment. Decreases in hatching size are commonly seen in metal toxicity tests [69], as Cu decelerates embryonic growth rates in many organisms, even at low concentrations [36]. Small hatching size is usually considered disadvantageous as it increases time in the plankton and therefore susceptibility to starvation and predation during later larval stages [70], but on the other hand may also confer advantages through enhanced dispersal, thus allowing larvae to escape local stressors [71]. In any case, hatching size was strongly affected by developmental treatment, showing that characteristics of planktonic *S*. *australis* larvae reflected environmental stressors experienced during development, and therefore highlighting a COE from one life stage to another.

No clear patterns based on parental treatment alone were found for hatching size, nor was there an effect on hatching time. Instead, COEs between life stages interacted with trans-generational COEs. For example, egg masses stressed with Cu had larger hatching larvae when laid by stressed adults. When parents were unstressed, developmental Cu exposure caused the smallest of all mean hatching sizes. Thus parental stress modified how embryos coped with exposure to Cu. Larger size may in this case be indicative of an anticipatory maternal effect (AME) that rendered offspring resistant to stress previously experienced by the parental generation and able to grow larger before hatching [72]. There is support for such an interpretation, as AMEs have been recorded in response to various pollutants in diverse aquatic taxa, including cadmium in fish [61], mercury in annelid worms [62], and Cu in gastropods [63]. Interaction between the two types of COE was widespread and the remainder of its consequences cannot easily be generalised.

By day 24, larvae from Cu-exposed egg masses showed opposite trends than at hatching, and were substantially smaller if their parents had been stressed as opposed to unstressed. This indicates that their initially large size at hatching may not in fact have conferred an advantage as they were overtaken in size by almost all other larvae during the rearing period, particularly Cu→Cu larvae, which hatched as some of the largest but ended their larval phase as the smallest. Thus results here closely mirror those in [64], where bryozoan offspring initially showed positive results in response to parental Cu exposure, but suffered a delayed performance reduction.

This research shows that both forms of COE (transgenerational and between life stages), each triggered by multiple global change-associated stressors, can interact to form highly context-dependent responses in an unstressed life stage of a marine organism. Because of their potential for far-reaching ecological consequences, it is likely that COEs will continue to gain recognition as major factors determining population and species persistence in the face of continued anthropogenic disturbances [20]. Ultimately, a deeper understanding of COEs may be critical for accurate predictions of how organisms will respond to ongoing global change.

## Supporting information

S1 Dataset The file summarizes all the relevant data that have been used in the statistical analyses. (XLSX)
